# Retrieval practice may not benefit mathematical word-problem solving

**DOI:** 10.3389/fpsyg.2023.1093653

**Published:** 2023-02-20

**Authors:** Xiaoxue Huang, Sining Zheng, Zhiyun Yu, Shunsen Chen

**Affiliations:** ^1^Fujian Key Laboratory of Applied Cognition and Personality, School of Educational Science, Minnan Normal University, Zhangzhou, China; ^2^School of Psychology, Fujian Normal University, Fuzhou, China; ^3^Institute of Marxism, Fujian Agriculture and Forestry University, Fuzhou, China

**Keywords:** retrieval practice effect, word-problem solving, example-problem pairs, mathematical, feedback

## Abstract

The retrieval practice effect refers to the fact that one or even multiple retrievals of memory content during the same period are more effective than repeated studying to promote future memory retention. It is effective for numerous declarative knowledge learning materials. However, studies have demonstrated that retrieval practice does not benefit problem-solving skill learning. This study used worked examples from math word problem tasks as learning materials, considering the retrieval difficulty as the main factor. Experiment 1 explored the effect of retrieval practice on acquiring problem-solving skills under different initial testing difficulties. Experiment 2 manipulated the difficulty of materials as a variable to ascertain the effect of retrieval practice on problem-solving skills under different material difficulty levels. Experiment 3 introduced feedback variables to facilitate the generation of the retrieval practice effect and examined the effects of various difficulty feedback levels on problem-solving skills learning. Results showed that, compared with restudying examples (SSSS), the example-problem pairs (STST) did not promote delayed test performance. As for the retrieval practice effect, as no differences or advantages were found in the repeated study group on the immediate test, the retrieval practice group generally outperformed the repeated study group on the delayed test. However, across the three experiments, we found no evidence of retrieval practice affecting results during an enhanced delayed test. Therefore, there may be no retrieval practice effect on acquiring problem-solving skills from worked examples.

## 1. Introduction

The retrieval practice effect demonstrates that one or even multiple retrievals of memory content during the same period are more effective than repeated studying in future memory content retention (Roediger and Karpicke, 2006b). The scope of the retrieval practice effect has not been fully clarified. Recent research has suggested that the benefits of retrieval practice are found in numerous declarative knowledge learning materials such as the free recall of word lists (Tulving, 1967), foreign language vocabulary (Carrier and Pashier, 1992; Karpicke and Roediger, 2008), paired-associate learning (Carpenter et al., 2008), anatomical facts (Grimaldi and Karpicke, 2014), scientific facts (McDermott and Naaz, 2014), GRE test preparation materials (Roediger and Karpicke, 2006a), text passages (Chan et al., 2006; Roediger and Karpicke, 2006b; Kang et al., 2007; Agarwal et al., 2008; Weinstein et al., 2010; Blunt and Karpicke, 2014), videos (Butler and Roediger, 2007; Cranney et al., 2009), and taxonomic vocabularies (Zaromb and Roediger, 2010). However, the application of retrieval practice to solve procedural knowledge problems in chemistry, mathematics, and physics is rarely addressed in practical educational and teaching activities.

Studies have demonstrated that retrieval practice does not benefit problem-solving skill learning (Van Gog and Kester, 2012; Leahy et al., 2015; Tran et al., 2015; Van Gog et al., 2015). Worked examples are important to establish, given the key role problem-solving plays in, for instance, math and science education. The so-called worked-example learning refers to a kind of learning method in which learners acquire problem-solving skills from examples that illustrate general principles, concepts, and procedures. Van Gog and Kester (2012) used examples (diagnosing circuit faults) as learning materials. A restudy examples group represented the group under the repeated studying condition (SSSS). An example-problem pairs is a study-test condition (STST), that is, a similar problem tackled immediately after studying the example (Kalyuga et al., 2001). Compared with the restudy examples group, the delayed test (1 week later) score of the example-problem pairs group (that is, the retrieval practice group) was not significantly higher than the immediate test, meaning that there was no retrieval practice effect. The three experiments of Leahy et al. (2015) also studied the application of retrieval practice on worked examples. Using worked examples as materials, primary school students were selected and divided into two groups: one for repeated study examples and the other for example-problem pairs. Experiments 1 and 2 showed that there was no retrieval practice effect on the immediate test (the performance of the simple restudy worked example was better than the example-problem pairs). As no differences or advantages were found in the repeated study group for the immediate test, the retrieval practice group generally outperformed the repeated studying group if the delayed test was used (Wheeler et al., 2003; Roediger and Karpicke, 2006a). Thus, Leahy et al. (2015) conducted Experiment 3, which used a delayed test with a one-week interval and still found no retrieval practice effect. Similarly, Van Gog et al. (2015) used worked examples (diagnosing circuit faults) as materials under initial test conditions (restudy example vs. example-problem pairs vs. example free recall), a final test (delayed vs. immediate + delay), and a final retention test (same vs. similar), without finding a retrieval practice effect.

Retrieval practice after a worked example study does not enhance delayed problem-solving performance compared to restudy. Learning from worked examples involves procedural knowledge; thus, for novices, it poses a high learning challenge, has a heavy cognitive load, and is difficult to understand. Therefore, the retrieval practice effect is promoted by reducing learning difficulty. There are two types of connection methods between examples and problems. In addition to the example-problem pairs, an incomplete example (that is, lack of some problem-solving procedures or steps, which need to be supplemented by the learner) may be added (Van Merrienboer et al., 1992). Studies have shown that incomplete examples can effectively support the acquisition of cognitive skills (Paas, 1992; Stark, 1999; Renkl, 2014). Furthermore, the quality of self-explanation in the incomplete example group was higher, and the problem-solving methods could be transferred. As the literature shows that incomplete examples have similar effects to learning examples on problem-solving tasks, they can be used as a form of testing. Similar to the two conditions of recall and recognition in the initial test, the initial test is different in form and difficulty, and the results will vary (Karpicke et al., 2014). Experiment 1 improves the research design of Van Gog et al. (2015) and explores the application of retrieval practice in worked examples. The improvement content is as follows. First, the selected materials are primary school mathematics Olympiad problems. Second, in the initial test conditions, the incomplete example condition is added to the two conditions of study example (restudy) and example-problem pairs (retrieval practice).

Paas and Van Merriënboer (1994) demonstrated that variation in examples promotes learning although other studies do not agree (Renkl et al., 1998). The participants in Experiment 1 studied similar examples rather than restudying the same material. If the similar materials used in Experiment 1 do not show a “pure” restudy condition, compared to the same materials (with the same structure and surface characteristics), the issue is whether the same materials can generate the retrieval practice effect in problem-solving. Simultaneous consideration of different materials (both structural and surface characteristics) may lead to varying results. Therefore, Experiment 2 introduces material variables, in which the same/similar/different materials refer to Example 2 being the same/similar/different from Example 1, while Example 4 being the same/similar/different from Example 3, that is, the same/similar/different problems were solved, and the example-problem pairs solved the same/similar/different problems as the learning example, except that the participants were required to solve the problems themselves.

In addition to reducing the difficulty, feedback can promote the retrieval practice effect (Carpenter and DeLosh, 2006; Kang et al., 2007; Butler and Roediger, 2008; Carpenter et al., 2008, 2009; Pyc and Rawson, 2010, 2012; Tse et al., 2010; Roediger et al., 2011; Putnam and Roediger, 2013; McDermott and Naaz, 2014). Giving feedback after acquiring problem-solving (retrieval practice condition) presents the problem-solving step and enables students to restudy examples of their problems, which resembles the restudy after an initial test. as research demonstrates an increased retrieval practice effect (Rawson and Dunlosky, 2012). Research on studying worked examples suggests that feedback/restudy can promote learning (Baars et al., 2014). Baars et al. (2014) asked students to learn example-problem pairs and then created a standard group (response feedback on correct problem-solving steps) to compare problem-solving performance with a non-standard group (no feedback on correct answers). They found that feedback helped with students’ self-assessment of performance and could improve the learning effect. In addition, Mullet et al. (2014) established that retrieval practice on problem-solving tasks resulted in better test scores following delayed feedback than immediate feedback. Nevertheless, none of the above research used restudy as a control condition (Baars et al., 2014; Mullet et al., 2014). Hence, Experiment 3 provides feedback to promote the generation of the retrieval practice effect in skill learning.

## 2. Experiment 1

Experiment 1 investigated the effect on the immediate and delayed retention test performance of the restudy example, example-problem pairs, and incomplete examples. As compared to the restudy example condition, the retrieval practice condition (example-problem pairs, incomplete example) required more mental effort and time (Bjork, 1994). So potential differences in invested mental effort across conditions were explored among the three initial test difficulties.

### 2.1. Methods

#### 2.1.1. Participants and design

The participants were 120 Minnan Normal University students (43 men; age *M* = 19.58, *SD* = 3.85) who did not take part in similar experiments before. They were randomly assigned to one of six conditions (*n* = 20 per condition) resulting from a 3 × 2 factorial design with between-subjects factors initial test conditions (restudy example, example-problem pairs, and incomplete example) and final test time (immediate, five mins; delayed, 1 week). The dependent variables were the final test scores and mental effort scores.

#### 2.1.2. Materials

Select Primary School Math Olympiad (Grade six) concepts were used as examples in the study. The learning conditions were divided into three groups: the restudying examples, the example-problem pairs, and the incomplete example. The learning material was four similar examples. “Similar materials” refer to the same structure but different surface features. It means that the problems described in the text are different, but the calculation steps are same (i.e., Example 1: A pasture is covered with grass, which grows at a uniform speed every day. This pasture can feed 10 cows for 20 days, or 15 cows for 10 days. How many days can 25 cows eat? Answer: suppose that the daily grass consumption of each cow is 1, (1) The daily growth rate of grass is: (10 * 20 − 15 * 10) / (20 − 10) = 5. (2) The initial grass amount is: (10 − 5) * 20 = 100. (3) Suppose 25 cows can eat for x days: (25 − 5) * x = 100, x = 5. Example 2: A piece of pasture has a certain stock of feed, and an equal amount of feed is purchased every day. Five sheep can eat the feed for 20 consecutive days, or six sheep can eat the feed for 15 consecutive days. If it is required to eat all the sheep in 6 days, how many sheep do you need at least? Answer: Suppose that each sheep eats 1 feed per day, (1) The rate of feed increase per day is: (5 * 20 − 6 * 15) / (20 − 15) = 2. (2) The original feed quantity is: (5 − 2) * 20 = 60. (3) How many sheep do you need at least? (x − 2) * 6 = 60, x = 12).

##### 2.1.2.1. Conceptual prior knowledge test

The conceptual prior knowledge test consisted of three open-ended questions about the primary school math Olympiad problem (“cow eating grass”). Before the experiment, each participant was tested for the conceptual prior knowledge level to exclude the influence of the participants’ original knowledge and experience regarding the experiment.

##### 2.1.2.2. Formula sheet

Explained the problem-solving ideas, principles, and corresponding formulas of the example (“cow eating grass” problem) [such as grass growth rate = (grazing speed 1 * time 1 − grazing speed 2 * time 2) / (time 1 − time 2)], presented separately *via* E-prime.

##### 2.1.2.3. Acquisition phase

In the acquisition phase, regarding the restudy example condition, Examples 1 and 2 on the one hand, and Examples 3 and 4 on the other, contained the same problem-solving steps. In the condition of the example-problem pairs, Examples 1 and 3 were for learning in the form of examples, while Examples 2 and 4 were for solving problems. In the incomplete example condition, Examples 1 and 3 were for learning in the form of examples, while Examples 2 and 4 were for the supplementing solving steps.

##### 2.1.2.4. Retention tests

The retention test comprised two questions, one of which was similar to what appeared in the initial test task (i.e., Q1: A ship has a leak. The water entered the ship at a uniform speed, and some water had already entered when the leak was found. If 12 people to pour water, they can finish it in 3 h; If only 5 people pour water, it will take 10 h to finish. How many people need to pour water in 2 h?); the other was different and would increase in difficulty, but both had come up before (i.e., Q2: There is a piece of grass on the pasture, which grows at a uniform speed every day. This pasture can feed 16 cows for 20 days, or 80 sheep for 12 days. If one cow eats as much grass as four sheep, how many days can 10 cows and 60 sheep eat together?).

##### 2.1.2.5. Mental effort rating scale

The participants were required to complete the Mental Effort Rating Scale after the acquisition phase and the retention tests. The scale was developed by Paas (1992) and was a 9-point scale ranging from (1) *very*, *very low effort* to (9) *very*, *very high effort*, to examine the problem-solving skills learning of diverse participant groups at the level of mental effort invested.

#### 2.1.3. Procedure

The experimental process was divided into the acquisition phase and the retention tests. Experiments were carried out in a quiet environment. Before the formal experiment, participants were asked to complete the conceptual prior knowledge test, and they were randomly assigned to each condition, then the computer presented the basic process and precautions of the experiment. In formal experiments, each group consisted of four similar examples. The computer presented each example individually for a maximum of 4 min per example.

##### 2.1.3.1. Acquisition phase

This phase was mainly for example study only. For the example study only, each participant group did not have to solve the problem alone, all the solution steps will be given, and the participants only needed to learn the steps. In the restudy examples condition, the participants continued to study the following example only after studying an example, until they finished four examples. For the example-problem pairs condition, after completing the example study only, the participants were presented with the problem of similar materials without a solution step, and required to answer on the answer sheet until they completed four examples. In the incomplete example condition, after completing the example study only (including the problem and specific problem-solving steps), the participants were presented with similar but incomplete examples (the problem-solving steps of the examples lacked the last step). The “incomplete problem” manipulation in Experiment 1 are shown below. At first, present an example. Then, the incomplete problem needed to be completed by the participants. There are three steps to solve the problem. Give the first two steps, and the third step needs to be completed by the participants themselves (i.e., Example 2: A piece of pasture has a certain stock of feed, and an equal amount of feed is purchased every day. Five sheep can eat the feed for 20 consecutive days, or six sheep can eat the feed for 15 consecutive days. If it is required to eat all the sheep in 6 days, how many sheep do you need at least? Answer: suppose that each sheep eats 1 feed per day, (1) The rate of feed increase per day is: (5 * 20 − 6 * 15) / (20 − 15) = 2. (2) The original feed quantity is: (5 − 2) * 20 = 60. (3) How many sheep do you need at least?). Participants complete the answers on the answer sheet until they completed four examples.

##### 2.1.3.2. Retention tests

The retention test had two questions, each was presented separately, and the participants needed to answer on the answer sheet. For the retention test answer score, each question consisted of three solution steps, three points for complete correctness, one and two points for partial correctness, and zero points otherwise. The points awarded based for the correct strategy use, considering the correct solution is reached for each step. As long as the strategy is correct, even if the result is wrong, points will be given. After completing the retention test, the participants in the immediate test were also required to perform a five-minute distraction task (watch a video); while those in the delayed test had to perform a distraction task. After a week, the participants on the delayed test took the retention test, while those on the immediate test completed a distraction task. The participants were required to rate their mental effort levels both during the study period and after the retention test.

### 2.2. Results

Through the conceptual prior knowledge test, the difference between the participants in each group did not reach a significant level, *F*(2,117) = 0.104, *p >* 0.05. In terms of retention test results, the main effect of different initial test conditions was not significant, *F*(2,118) = 0.493, *p* = 0.612, η_p_^2^ = 0.008; that is, the difference in the scores of the three conditions of restudy example, example-problem pairs, and the incomplete example did not reach a significant level. Meanwhile, the main effect of different test times was also not significant, *F*(1,118) = 0.042, *p* = 0.837, η_p_^2^ = 0.000; that is, the participants in the immediate retention test and the delayed retention test were not significantly different. The interaction difference between the initial test conditions and test time did not reach a significant level, *F*(2,118) = 0.332, *p* = 0.718, η_p_^2^ = 0.006 (Table 1).

**Table 1:** The effect of retrieve practice on problem-solving under different initial test conditions.

	Immediate retention test	Delayed retention test
Example-problem pairs	3.75(2.31)	3.35(2.34)
Restudy example	3.50(1.93)	3.94(2.22)
Incomplete example	4.00(2.16)	4.11(2.17)

In terms of the degree of mental effort, the main effect of different testing times was not significant, *F*(1,118) = 2.28, *p* = 0.134, η_p_^2^ = 0.019. The main effect of different initial test conditions was significant, *F*(2,118) = 3.66, *p* = 0.029, η_p_^2^ = 0.059. The results of the LSD multiple comparisons revealed that the difference between the incomplete example and the example-problem pairs reached a significant level. The interaction difference between the initial test conditions and test time did not reach a significant level, *F*(2,118) = 2.888, *p* = 0.06, η_p_^2^ = 0.048 (Table 2).

**Table 2:** The mental effort of retrieve practice on problem-solving under different initial test conditions.

	Immediate retention test	Delayed retention test
Example-problem pairs	7.40(1.18)	5.71(1.73)
Restudy example	6.40(1.75)	6.32(1.66)
Incomplete example	5.70(1.76)	5.76(2.01)

### 2.3. Discussion

Experiment 1 found that there was no retrieval practice effect. Compared with the restudy example group, the difference in retention test scores (immediate or delayed test) was insignificant, whether it was the example-problem pairs or the incomplete example group. The results were consistent with previous findings (Van Gog et al., 2011).

Van Gog and Kester (2012) discovered a significant difference between the example-problem pairs group and the restudy example group in the delayed retention test. However, these outcomes did not align with those from our experiment. It may be due to Van Gog and Kester's (2012) test time variable using a within-group design; that is, the process of immediate retention test affected subsequently delayed test scores. Our study employed a between-group design to test the time variable. Meanwhile, similar examples used in this study as experimental materials may result in different experimental results. In this experiment, the restudy example group learned similar examples during the acquisition phase; therefore, the variability of examples may have facilitated learning compared to restudying identical examples (Paas and Van Merriënboer, 1994).

Moreover, the incomplete example group was added to the initial test difficulty of this experiment. The incomplete example group and the example-problem pair group belong to the two example-problem connection methods, and both belong to the problem-solving group (retrieve practice group). However, the former belongs to the hierarchical level task, while the latter belongs to the whole level task. While both belong to the problem-solving method, their difficulty in solving the problem is different—the former is weaker, and the latter is stronger. Incomplete examples require the learner to complete/supplement solving steps (i.e., a decreasing strategy, Paas, 1992; Renkl and Atkinson, 2003). This problem-solving style is viewed as a hierarchical task in the test style and is effective in acquiring problem-solving skills from examples (Renkl, 2014). However, its effect on the delayed retention test was less studied compared to the restudy example or the example-problem pairs. Our study found that in terms of the difficulty level of problem-solving tasks, compared with the example-problem pairs, the incomplete example had lower retrieval difficulty, while there was no retrieval practice effect. Nevertheless, the effect of this retrieval practice (hierarchical task) might be superior to those of the complete task.

For the input of mental effort, the main effect of different initial test difficulties was significant; that is, there was a large difference in effort between the example-problem pairs and the incomplete example. As such, the effort input under the incomplete example condition was significantly lower than that of the example-problem pairs condition. It means the example-problem pairs require the highest mental effort. The incomplete example condition and the example-problem pairs condition were both parts of the retrieval practice. However, they brought about notable differences, which may be due to the complexity of the task (the participants could not fully understand the learning task) or the different participant motivations.

## 3. Experiment 2

Paas and Van Merriënboer (1994) demonstrated that variation in examples promotes learning although other studies do not agree (Renkl et al., 1998). The participants in Experiment 1 studied similar examples rather than restudying the same material. If the similar materials used in Experiment 1 do not show a “pure” restudy condition, compared to the same materials (with the same structure and surface characteristics), the issue is whether the same materials can generate the retrieval practice effect in problem-solving. Simultaneous consideration of different materials (both structural and surface characteristics) may lead to varying results.

The purpose of Experiment 2 was to examine the effect of retrieval practice on problem-solving under different material conditions. It also explored possible differences in mental effort input under each condition.

### 3.1. Methods

#### 3.1.1. Participants

The participants were 120 Minnan Normal University students (33 men; age *M* = 19.98, *SD* = 3.45) who did not participate in similar experiments before. They were randomly assigned to one of the conditions. However, because some participants did not continue to participate in the delayed retention test, so there were only 111 participants left: different material example-problem pairs group (*n* = 18), the different materials restudy example group (*n* = 18), the same materials restudy example group (*n* = 16), similar materials example-problem pairs (*n* = 19), same materials example-problem pairs and similar materials restudy example groups (*n* = 20).

#### 3.1.2. Materials

The materials are identical to those used in Experiment 1, except for the acquisition phase learning materials with different properties. The learning material consisted of four examples that were the same, similar, or different. “Same materials” refers to the same structure and surface features (overview, numbers); “similar materials” refers to the same structure but different surface features; “different materials” refers to the variability of examples with dissimilar structures and surface features. It does not mean any example, but also belongs to the “cow eating grass” problem. The different examples are as follows: the Example 1 and 2. They have different structures and surface, but all belong to the “cow eating grass” problem. Example 1: A pasture is covered with grass, which grows at a uniform speed every day. This pasture can feed 10 cows for 20 days, or 15 cows for 10 days. How many days can 25 cows eat? Answer: suppose that the daily grass consumption of each cow is 1, (1) The daily growth rate of grass is: (10 * 20 − 15 * 10) / (20 − 10) = 5. (2) The initial grass amount is: (10 − 5) * 20 = 100. (3) Suppose 25 cows can eat for x days: (25 − 5) * x = 100, x = 5. Example 2: As the weather gets colder, the grass on the pasture decreases at a uniform speed every day. The grass can feed 20 cows for 5 days, or 16 cows for 6 days. How many days can 11 cows eat? Answer: suppose that the daily grass consumption of each cow is 1. (1) The rate of grass reduction per day is: (20 * 5 − 16 * 6) / (6 − 5) = 4. (2) The initial grass amount is: (20 + 4) * 5 = 120. (3) Suppose 11 cows can eat for x days? (11 + 4) * x = 120, x = 8. In the acquisition phase, regarding the restudy example condition, if the same material is used, then Examples 1 and 2, on the one hand, as well as Examples 3 and 4, on the other contain the same problem-solving steps. If similar or different materials were selected, Examples 1 and 2, on the one hand, as well as Examples 3 and 4, on the other, contained similar or different problem-solving steps. In the example-problem pairs condition, Examples 1 and 3 were for learning in the form of examples, while Examples 2 and 4 were for solving problems. The steps for solving the problem in Examples 2 and 4 are the same if the same materials were utilized as in Examples 1 and 3, respectively. If similar or different materials were selected, the problem-solving steps of Example 2 were similar or different from those of Example 1, and those of Example 4 were similar or different from those of Example 3.

#### 3.1.3. Design

A mixed experimental design of 2 (initial test conditions: restudy example, example-problem pairs) × 3 (material: similar, same, different) × 2 (test time: immediate, delayed) was adopted. The initial test conditions and materials were manipulated a between-subjects design, and the testing time was manipulated a within-subjects design. The dependent variables were the final test scores and mental effort scores.

#### 3.1.4. Procedure

The procedure was identical to that used in Experiment 1, except the acquisition phase removed the incomplete example and learned materials with different properties. Acquisition phase: this phase was mainly to learn about similar, same, or different examples. For studying examples only, each group of participants did not have to solve the problem alone, all the solution steps would be given, and the participants only needed to learn the steps. For the same materials, in the restudying example condition, the participants continued to study the same examples after studying the example, until they had studied four examples; In the example-problem pair condition, after the example was studied, the problem of the same materials (with no solution steps) were presented, and the participants responded on the answer sheet until they were completed. The same process was followed for similar materials and different materials.

### 3.2. Results

For conceptual prior knowledge level, the difference between the participants in each group did not reach a significant level, *F*(2,107) = 0.355, *p >* 0.05. Repeated measures analysis of variance was performed on initial learning conditions × material properties × testing time. In terms of final scores, the main effect of learning conditions was not significant, *F*(1,107) = 0.735, *p* = 0.393, η_p_^2^ = 0.007; the main effect of material properties was not significant, *F*(2,107) = 2.13, *p* = 0.124, η_p_^2^ = 0.038; the main effect of test time was significant, *F*(1,107) = 5.24, *p* = 0.024, η_p_^2^ = 0.047, that is, the participant’s performance on the immediate test was significantly lower than the delayed test; the interaction between learning conditions and test time did not reach a significant level, *F*(1,107) = 1.378, *p* = 0.243, η_p_^2^ = 0.013, the interaction difference between material properties and testing time did not reach a significant level, *F*(2,107) = 0.532, *p* = 0.589, η_p_^2^ = 0.01. The interaction between learning conditions and material properties did not reach a significant level, *F*(2,107) = 1.442, *p* = 0.241, η_p_^2^ = 0.027. The interaction of three variables did not reach a significant level, *F*(2,107) = 1.953, *p* = 0.147, η_p_^2^ = 0.036 (Table 3).

**Table 3:** The effect of retrieve practice on problem-solving under different material properties.

	Immediate retention test			Delayed retention test		
	Different material	Same material	Similar material	Different material	Same material	Similar material
Example-problem pairs	3.89(2.32)	3.15(1.76)	3.95(2.19)	4.33(2.47)	4.40(1.67)	4.53(1.93)
Restudy example	5.11(1.78)	4.06(1.95)	3.50(1.93)	5.17(1.65)	3.63(2.34)	4.50(2.24)

For mental effort level, the main effect of the learning conditions was not significant, *F*(1,107) = 0.003, *p* = 0.957, η_p_^2^ = 0.00; the main effect of the material property was also not significant, *F*(2,107) = 0.49, *p* = 0.614, η_p_^2^ = 0.009; the main effect of test time was significant, *F*(1,107) = 4.312, *p =* 0.04, η_p_^2^ = 0.039, that is, the mental effort of the participants in the immediate test was significantly higher than that in the delayed test; the interaction difference between learning conditions and test time did not reach a significant level, *F*(1,107) = 1.435, *p* = 0.234, η_p_^2^ = 0.013; the interaction of material properties and test time did not differ significantly, *F*(2,107) = 1.68, *p* = 0.191, η_p_^2^ = 0.03. The interaction between learning conditions and material properties reach a significant level, *F*(2,107) = 4.409, *p* = 0.015, η_p_^2^ = 0.077. The similar materials reach a significant level, *p* = 0.021. The interaction of three variables did not reach a significant level, *F*(2,107) = 1.055, *p* = 0.352, η_p_^2^ = 0.02 (Table 4).

**Table 4:** The mental effort of retrieve practice on problem-solving under different material properties.

	Immediate retention test			Delayed retention test		
	Different material	Same material	Similar material	Different material	Same material	Similar material
Example-problem pairs	6.00(2.25)	5.80(1.36)	7.47(1.17)	6.11(1.45)	6.05(1.70)	6.63(1.64)
Restudy example	6.67(1.71)	7.00(1.41)	6.40(1.76)	6.50(1.34)	6.13(1.50)	5.65(2.06)

### 3.3. Discussion

Experiment 2 demonstrated that there was no retrieval practice effect in studying examples under the three material properties. Potentially, retrieval practice is ineffective in promoting long-term memory storage for acquiring problem-solving materials. It may be because the participants did not fully understand or successfully answer the questions (although it involved studying the same examples).

The final scores and mental effort input had significant main effects on test time. The immediate test scores were significantly lower than those under the delayed test, and the mental effort input in the immediate test was significantly higher than that under the delayed test. It indicates that the final scores and mental effort may be inversely proportional; that is, the higher the performance, the lower the mental effort input.

In terms of difficulty, different materials were more challenging than similar materials, which were more difficult than the same materials. Theoretically, the same materials should have the best scores, and the different materials would be the worst; however, in some cases, particularly when restudying, the opposite may be true. In contrast to the example-problem pairs condition, the participants under the restudy example condition frequently overestimated their performance and did not engage in continuous study, which led to lower scores. However, due to its higher material difficulty, the restudy example under different materials may enhance the participants’ learning motivation.

## 4. Experiment 3

The purpose of Experiment 3 was to examine the effect of retrieval practice on acquiring problem-solving skills under different feedback times. As retrieving could be assumed to be more effortful than restudying, potential differences in the mental effort were explored.

### 4.1. Methods

#### 4.1.1. Participants

The participants were 80 Minnan Normal University students (20 men, age *M* = 20.98, *SD* = 3.45) who had not engaged in similar experiments before. They were randomly assigned to any of the four conditions (*n* = 20). As the participants under some conditions did not take part in the delayed test 1 week later, group size varied as follows: the immediate feedback example-problem pairs group (*n* = 12), the immediate feedback restudy examples group (*n* = 19), the delayed feedback example-problem pairs group (*n* = 14), the delayed feedback restudy examples group (*n* = 17).

#### 4.1.2. Materials

The materials were identical to those used in Experiment 1.

#### 4.1.3. Design

A mixed experimental design of 2 (initial test conditions: restudy example, example-problem pairs) * 2 (feedback time: immediate, delayed) * 2 (test time: immediate, delayed) was adopted. The initial test conditions and feedback time used a between-subjects design, and the testing time was manipulated a within-subjects design. The dependent variables were the final test scores and mental effort scores.

#### 4.1.4. Procedure

The procedure was identical to that used in Experiment 1, with the following exception: removed the incomplete example condition and added feedback (immediate, delayed) during the acquisition phase.

Acquisition phase: This stage was mainly to learn from similar examples. For study only, the participants in each group did not have to try to solve the problem by themselves, all the solution steps were given, and they merely had to learn the steps. In the example-problem pair condition, if immediate feedback was given, after the participants had answered Examples 2 and 4, they presented all the steps to solve the problem, respectively; that is, the material presentation method was Example 1 − Example 2 − Feedback (The problem-solving steps of Example 2) − Example 3 − Example 4 − Feedback (The problem-solving steps of Example 4) until four examples were completed. The presentation time of feedback (problem-solving steps) was 1 min. Then they took the immediate test and a delayed test a week later. If delayed feedback was given, it was after completing the four examples, taking part in the instant test, and giving feedback a week later. This process occurred when the problem-solving steps of Examples 2 and 4 were presented completely, and the presentation time was 1 min, respectively, and followed by the delayed test. In the restudy examples condition, the participants continued to study similar examples after studying an example, until they finished four examples; if immediate feedback was given, to maintain balance, the participants only study were extended the study time. The total time was the same for both study conditions. Then they would take the immediate test and a delayed test a week later. If delayed feedback was given, it was after completing four examples, participating in the immediate test, and receiving feedback a week later. They then restudied Examples 2 and 4, and the presentation time was 1 min, respectively, and subsequently, they took part in the delayed test again.

### 4.2. Results

Through the survey of conceptual prior knowledge level, there was no significant difference among the participants in each group, *F*(2,59) = 0.075, *p >* 0.05. An ANOVA was performed on the learning conditions, feedback, and test time. In terms of the final score, the main effect of the learning conditions was not significant, *F*(1,59) = 0.406, *p* = 0.527, η_p_^2^ = 0.007; the main effect of feedback was not significant, *F* (1,59) = 0.31, *p* = 0.58, η_p_^2^ = 0.005; the main effect of test time was also not significant, *F*(1,59) = 1.725, *p* = 0.194, η_p_^2^ = 0.028; the interaction difference between learning conditions and test time was not significant, *F*(1,59) = 0.976, *p* = 0.327, η_p_^2^ = 0.016; the interaction difference between the feedback and test time was not significant, *F*(1,59) = 2.967, *p* = 0.09, η_p_^2^ = 0.048. The interaction between the learning conditions and the feedback did not reach a significant level, *F*(1,59) = 0.307, *p* = 0.582, η_p_^2^ = 0.005. The interaction of three variables did not reach a significant level, *F*(1,59) = 3.964, *p* = 0.051, η_p_^2^ = 0.064 (Table 5).

**Table 5:** The effect of the different difficulties of retrieval feedback on problem-solving.

	Immediate retention test		Delayed retention test	
	Immediate feedback	Delayed feedback	Immediate feedback	Delayed feedback
Example-problem pairs	4.92(1.62)	4.57(1.91)	4.50(1.78)	3.86(2.14)
Restudy example	4.68(1.33)	3.76(2.01)	3.74(2.46)	4.59(1.58)

For the level of mental effort, the main effect of the learning conditions was not significant, *F*(1,59) = 0.071, *p* = 0.0791, η_p_^2^ = 0.001; the main effect of feedback was also not significant, *F*(1,59) = 0.023, *p* = 0.879, η_p_^2^ = 0.000; the main effect of test time not significant, *F*(1,59) = 1.489, *p* = 0.227, η_p_^2^ = 0.025; the interaction between learning conditions and test time did not reach a significant level, *F*(1,59) = 0.573, *p* = 0.452, η_p_^2^ = 0.01; the interaction between the feedback and the test time was not significant, *F*(1,59) = 1.345, *p* = 0.251, η_p_^2^ = 0.022. The interaction between the learning conditions and the feedback did not reach a significant level, *F*(1,59) = 0.043, *p* = 0.836, η_p_^2^ = 0.001. The interaction of three variables did not reach a significant level, *F*(1,59) = 0.27, *p* = 0.606, η_p_^2^ = 0.005 (Table 6).

**Table 6:** The mental effort of different difficulties of retrieval feedback on problem-solving.

	Immediate retention test		Delayed retention test	
	Immediate feedback	Delayed feedback	Immediate feedback	Delayed feedback
Example-problem pairs	5.0(2.63)	5.43(1.65)	6.0(1.76)	5.50(1.45)
Restudy example	5.16(2.11)	5.47(1.46)	5.47(1.61)	5.41(1.77)

### 4.3. Discussion

Experiment 3 further found that there was no retrieval practice effect under different feedback difficulty levels on problem-solving skills learning. The results in Experiments 1 and 2 may be explained by the fact that the retrieval practice after the studying example process during the acquisition phase cannot generate the retrieval practice effect, and the studying example process (procedural knowledge acquisition) was more inclined to have its characteristics. The example effect of this knowledge system is structured, non-declarative knowledge (without structural association before and after), and thus, creates the opposite result of the retrieval practice effect (under the condition of delayed feedback). However, it did not occur under the condition of immediate feedback, which may be because the difficulty of retrieval of immediate feedback is lower than that of delayed feedback. Furthermore, the experiment used similar materials, and the combination of the two feedback types did not generate the effect of retrieval practice.

Studies have shown that feedback contributes to the retrieval practice effect (Carpenter et al., 2008, 2009; McDermott and Naaz, 2014). For those with no previous retrieval practice effect, giving delayed feedback can promote this effect (Pyc and Rawson, 2010, 2012; Tse et al., 2010; Roediger III et al., 2011; Putnam and Roediger, 2013). Although interpreted from its definition, the so-called retrieval practice effect can be obtained even when testing is performed without feedback (Carrier and Pashier, 1992). However, when the effect of retrieval practice cannot be obtained, giving feedback can promote its generation; additionally, adding feedback after retrieval practice can further promote long-term memory retention on the original basis. However, the reason why retrieval practice improves learning is that it can be successfully retrieved. If the tester does not retrieve the correct answer and does not restudy it, it will adversely affect the retrieval practice effect (Roediger and Marsh, 2005). Therefore, the results of Experiment 3 may also correlate with whether the participants actually understood or successfully retrieved the examples.

## 5. General discussion

The three experiments delineated above jointly find that the retrieval practice effect may not apply to acquiring problem-solving skills. The restudy examples only were used for repeat studying. The example-problem pairs with only questions and no problem-solving step results were used as retrieval practice, that is, the participants were asked to retrieve content. This study was mainly carried out by retrieving three aspects of the difficulty of practice, namely, initial test difficulty, material difficulty, and feedback difficulty. The difficulty of the initial test was divided into learning conditions: restudy example, example-problem pairs, and incomplete example. The difficulty of materials was divided according to the properties of materials: similar, same, and different. The difficulty of feedback was divided based on timing: immediate feedback (after 5 min) and delayed feedback (after 1 week). Through a step-by-step experiment, it was found that the retrieval practice effect cannot be realized in the example.

As the students were primarily in the cognitive framework or mode of the learning step procedure during the study of the example, they appeared to benefit less from the learning effects of problem-solving. Recent studies have shown that the combination of studying examples and problem-solving yields little benefit (Baars et al., 2014; Van Gog et al., 2015). Baars et al. (2014) mentioned that students who learned the right problems from the three examples did not perform better than those who studied the three examples, and the advantage of problem-solving learning is that it helps students to self-assess their performance more accurately. Problem-solving learning has fewer overestimated test scores than learning by example. Therefore, perhaps the example retrieval practice does not promote learning or memory retention, but it may enhance the accuracy of students’ metacognition or motivation and the persistence of learning behavior.

Regarding the mental effort input, Experiment 1 demonstrated that under the immediate test, the example-problem pairs group was more invested than the incomplete examples group, and the difference was significant. Studies have established that the effort value of example-problem pairs will be more than that of restudy examples (Van Gog et al., 2015). However, few studies on mental effort input use incomplete examples. Thus, the example-problem pairs group may be motivated significantly more than the incomplete examples group. The reason may be that students are not aware of whether they have successfully mastered this skill during the repeated restudy example process; therefore, they may overestimate themselves and their mental effort will be reduced. In the process of retrieving, students in the example-problem pairs group were constantly trying to solve problems; thus, they could make more objective self-judgments (metacognition), and more mental effort would be invested. The incomplete examples group is similar to the restudy examples group in terms of mental effort. While Experiment 2 confirmed this fact, the differences in Experiment 3 did not reach a significant level. In addition, Experiment 2 showed that the students’ scores were inversely proportional to their mental effort input; on the contrary, this result was not obvious in Experiments 1 and 3.

Restudying/feedback after testing, according to Rawson and Dunlosky (2012), can boost the retrieval practice effect. Our experiments, however, were not able to confirm this aspect. However, it has not been verified in the examples. Recent studies suggested that restudy/feedback may promote examples of studying (Baars et al., 2014). Baars et al. (2014) compared grades by having students examine example-problem pairs with no feedback and standard feedback (correct answers for each step). The results demonstrated that providing feedback to students increased their learning outcomes and ability to self-evaluate their grades (compared to no standard feedback). However, no real restudy was involved in the research. It is, therefore, unknown whether feedback (not having standard answers or restudying after a test) can improve scores. The study has also shown that feedback following problem-solving may just lengthen learning time rather than achieving learning outcomes (McLaren et al., 2014). Therefore, the real effect of feedback in Experiment 3 cannot be effectively defined.

The failure of the retrieval practice effect on the example study may be relatively due to the short acquisition phase (Van Gog et al., 2015). The participants probably believed that practicing the problem-solving procedure was more effective than studying when analyzing the effect of retrieval practice (compared to the restudy group) during the long study period. Therefore, the retrieval practice effect (compared to restudy) obtained in a long sequence task requires further study, whether it is the retrieval practice effect or the product of advanced guidance, that is, studying examples reaches a certain level and the participant’s knowledge level reaches a point, gaining the ability to solve problems on their own (no need to examples study; Kalyuga et al., 2001, 2003; Kalyuga, 2007).

Provided the critical prompt in the acquisition phase, retrieval practice enhanced analogical problem-solving (Hostetter et al., 2019). However, Peterson and Wissman (2018) revealed no effect of retrieval practice on analogical problem-solving. They discussed the impact of learning styles on analogical problem solving, using the same learning materials, but got varying results. The difference was that the prompt appeared in the acquisition phase or the test period. Their investigation revealed that the position of the prompt was very important. Eglington and Kang (2018) reported that retrieval practice could improve deductive inference through the presentation format of the material. The material format and prompt could enhance the accuracy of initial learning. Only by ensuring the success rate of initial retrieval can participants benefit from retrieval (Karpicke et al., 2014).

The key takeaway from our study is that procedural knowledge, one component of skill knowledge acquisition, produces the least significant effects. According to current research, this type of information, which is more closely related to working memory than declarative knowledge, was highly correlated with context or structure. However, neither the division of procedural knowledge nor the division of experimental materials has an operational definition. There are no quantitative measures, even though Van Gog and Sweller (2015) classified “complexity” and “material component interaction” as low, medium, and high. It is therefore necessary to quantify these aspects in future studies.

## Data availability statement

The raw data supporting the conclusions of this article will be made available by the authors, without undue reservation.

## Ethics statement

The studies involving human participants were reviewed and approved by the Academic Committee of Minnan Normal University. The patients/participants provided their written informed consent to participate in this study.

## Author contributions

XH conceived and designed this study, collected and analyzed the data, and wrote and revised the manuscript. SZ, ZY, and SC revised the manuscript. XH and SC were responsible for the study. All authors contributed to the article and approved the submitted version.

## Funding

This research was supported by the National Office for Education Sciences Planning, Ministry of Education of the People’s Republic of China through Grant BBA220203.

## Conflict of interest

The authors declare that the research was conducted in the absence of any commercial or financial relationships that could be construed as a potential conflict of interest.

## Publisher’s note

All claims expressed in this article are solely those of the authors and do not necessarily represent those of their affiliated organizations, or those of the publisher, the editors and the reviewers. Any product that may be evaluated in this article, or claim that may be made by its manufacturer, is not guaranteed or endorsed by the publisher.
