# Supplementary material for: Retrieval practice may not benefit mathematical word-problem solving
Source: Front Psychol. 2023 Feb 20;14:1093653. doi: 10.3389/fpsyg.2023.1093653 (PMC9987560; doi:10.3389/fpsyg.2023.1093653)
Supplement: Supplementary file 1 [file Data_Sheet_1.PDF]

相似、相同、不同样例的样例

一、下面是样例问题对组的相似性样例：

英国科学家牛顿提出了“牛吃草”问题，即牛在牧场上吃草，牧场上的草在不断的、均匀的生长。同一片牧场中的“牛吃草”问题，其**公式**为：

草生长速度=（吃草速度1*时间1-吃草速度2*时间2）/（时间1-时间2）

初始草量=（吃草速度-草生长速度）*时间

学习并记忆上面公式，学习完毕请**点击鼠标**

例1：

一片牧场长满牧草，牧草均匀生长。这片牧草可供10头牛吃20天，可供15头牛吃10天。则可供25头牛吃几天？

答：设每头牛每天吃草量为1，

（1）每天草的生长速度是：

（10*20-15*10）/(20-10)=5

（2）初始草量是：

20*（10-5）=100

（3）设可供25头牛吃x天：

x*（25-5）=100，x=5

例2：

一片牧场原有库存饲料一定，而且每天都购进相等数量的饲料入库。5只羊连续20天可吃完饲料，6只羊连续15天可吃完。若要求在6天里正好全部吃完，则至少需要几只羊？

请在答题纸上进行解答，时间为**4分钟**！

例3：

某篮球比赛14:00开始，13:30允许观众入场，但早有人来排队等候入场，假设从第一个观众来到时起，每分钟来的观众人数一样多，如果开3个入场口，13:45就不再有人排队；如果开4个入场口，13:40就没有人排队；那么第一个观众到达的时间是几点？

答：设每个入场口每分钟入场的观众为1，

（1）每分钟来的观众数量为：

（3*15-4*10）/(15-10)=1

（2）最初的观众数量为：

15*（3-1）=30

（3）那么第一个观众到达的时间为：

观众进入会场所用时间为30/1=30分，13:30分往前推30分钟第一个观众到达，即为13:00。

例4：

画展9点开门，但早有人来排队入场，从第一个观众来到时起，若每分钟来的观众一样多，如果开3个入场口，9点9分就不再有人排队；如果开5个入场口，9点5分就没有人排队。求第一个观众到达的时间？

请在答题纸上进行解答，时间为**4分钟**！

二、下面是样例问题对组的相同性样例：

例1：

一片牧场长满牧草，牧草均匀生长。这片牧草可供10头牛吃20天，可供15头牛吃10天。则可供25头牛吃几天？

答：设每头牛每天吃草量为1，

（1）每天草的生长速度是：

(10*20-15*10)/(20-10)=5

（2）初始草量是：

20*(10-5)=100

（3）设可供25头牛吃x天：

x*(25-5)=100，x=5

例2：

一片牧场长满牧草，牧草均匀生长。这片牧草可供10头牛吃20天，可供15头牛吃10天。则可供25头牛吃几天？

请在答题纸上进行解答，时间为4分钟！

例3：

某篮球比赛14:00开始，13:30允许观众入场，但早有人来排队等候入场，假设从第一个观众来到时起，每分钟来的观众人数一样多，如果开3个入场口，13:45就不再有人排队；如果开4个入场口，13:40就没有人排队；那么第一个观众到达的时间是几点？

答：设每个入场口每分钟入场的观众为1，

（1）每分钟来的观众数量为：

(3*15-4*10)/(15-10)=1

（2）最初的观众数量为：

15*(3-1)=30

（3）那么第一个观众到达的时间为：

观众进入会场所用时间为30/1=30分，13:30分往前推30分钟第一个观众到达，即为13:00。

例4：

某篮球比赛14:00开始，13:30允许观众入场，但早有人来排队等候入场，假设从第一个观众来到时起，每分钟来的观众人数一样多，如果开3个入场口，13:45就不再有人排队；如果开4个入场口，13:40就没有人排队；那么第一个观众到达的时间是几点？

请在答题纸上进行解答，时间为4分钟！

三、下面是样例问题对组的不同性样例：

例1：

一片牧场长满牧草，牧草均匀生长。这片牧草可供10头牛吃20天，可供15头牛吃10天。则可供25头牛吃几天？

答：设每头牛每天吃草量为1，

（1）每天草的生长速度是：

（10*20-15*10）/(20-10)=5

（2）初始草量是：

20*（10-5）=100

（3）设可供25头牛吃x天:

x*（25-5）=100，x=5

例2：

由于天气逐渐变冷，牧场上的草每天以均匀的速度减少。经计算，牧场上的草可供20头牛吃5天，或可供16头牛吃6天。那么，可供11头牛吃多少天？

请在答题纸上进行解答，时间为4分钟！

例3：

由于天气干旱，村委会决定用抽水机抽取水库中剩余的水浇灌农田。加入每天水库的水以均匀的速度增发，经计算，若用20台抽水机全力抽水，水库中水可用5周；若用16台抽水机，水库中水可用6周；若用11台抽水机，水库中的水可用多少周？

答：设每台抽水机每周抽水量为1份，

（1）设每周蒸发量为x：

20*5+5x=16*6+6x，x=4

（2）水库的初始水量为：

20*5+5*4=120

（3）设水库中的水可用x周：

x*（11+4）=120，x=8

例4：

有三块草地，面积分别为4公顷、8公顷和10公顷，草地上的草一样厚，而且长得一样快。第一块草地可供24头牛吃6周，第二块草地可以供36头牛吃12周。问：第三块草地可供50头牛吃几周？

请在答题纸上进行解答，时间为4分钟！

附录二 自编问卷

非常感谢参加本次实验！在完成基本信息以及第一部分的填写后，请您认真遵照指导语操作即可，如有不明白的可在咨询完我们的工作人员后再进行。

基本信息

姓名：_______ 专业：_______ 年龄：________ 学历：________ 年级：________

性别：_______ 高考数学分数：[<90 分, 90-110 分,110-130 分，>130 分]

第一部分

1、您知道“牛吃草”问题吗？________________

2、（选做）如果知道，是从哪里了解的？_____________________________

3、（选做）您熟悉“牛吃草”问题的计算公式吗？请按熟悉程度给分，1 分为非常不熟悉，5 分为非常熟悉。

1 2 3 4 5

请停止阅读下文，遵循电脑指示后，方可进入完成接下来的几个部分！！！

第二部分 学习部分

公式栏

英国科学家牛顿提出了“牛吃草”问题，即牛在牧场上吃草，牧场上的草在不断的、均匀的生长。同一片牧场中的“牛吃草”问题，其公式为：

$$草生长速度=\frac{（吃草速度1*时间1-吃草速度2*时间2）}{（时间1-时间2）}$$

初始总草量=（吃草速度-草生长速度）*时间

（1）例 2 (可参考公式栏公式）

答：

注：反面还有题目！！

例 4 (可参考公式栏公式）

答：

（2）心理努力等级评定

请对上述学习内容进行心理努力九级评定，即你所投入的解决问题的心理努力值，其中 1-9 分别代表 1、非常、非常低努力；2、非常低努力；3、低努力；4、稍低努力；5、一般努力；6、稍高努力；7、高努力；8、非常高努力；9、非常、非常高努力。

1 2 3 4 5 6 7 8 9

第三部分 正式测验

（1）题 1 (可参考公式栏公式）

答：

题 2 (可参考公式栏公式）

答：

（2）心理努力等级评定

请对上述学习内容进行心理努力九级评定，即你所投入的解决问题的心理努力值，其中 1-9 分别代表 1、非常、非常低努力；2、非常低努力；3、低努力；4、稍低努力；5、一般努力；6、稍高努力；7、高努力；8、非常高努力；9、非常、非常高努力。

1 2 3 4 5 6 7 8 9
