# Supplementary material for: Retrieval practice may not benefit mathematical word-problem solving
Source: Front Psychol. 2023 Feb 20;14:1093653. doi: 10.3389/fpsyg.2023.1093653 (PMC9987560; doi:10.3389/fpsyg.2023.1093653)
Supplement: Supplementary file 2 [file Data_Sheet_2.pdf]

Appendix 1 Examples of Similar, Same and Different Instances

British scientist Newton proposed "the problem of cow grazing", that is, cows graze on the pasture, and the grass on the pasture grows continuously and uniformly. The "cow grazing" problem in the same pasture is formulated as:

$$\text{Grass growth rate} = \frac{(\text{grazing speed } 1 \times \text{time } 1 - \text{grazing speed } 2 \times \text{time } 2)}{(\text{time } 1 - \text{time } 2)}$$

$$\text{Initial grass amount} = (\text{grazing speed} - \text{grass growth speed}) \times \text{time}$$

Study and memorize the above formula, please click the mouse after studying!

Similar Examples in Example-problem Pairs Condition

Example 1:

A pasture is covered with grass, which grows at a uniform speed every day. This pasture can feed 10 cows for 20 days , or 15 cows for 10 days. How many days can 25 cows eat?

Answer: Suppose that the daily grass consumption of each cow is 1,

(1)The daily growth rate of grass is: (10*20-15*10)/(20-10)=5.

(2)The initial grass amount is: (10-5) * 20=100.

(3)Suppose 25 cows can eat for x days: (25-5) * x=100,x=5.

Example 2:

A piece of pasture has a certain stock of feed, and an equal amount of feed is purchased every day. Five sheep can eat the feed for 20 consecutive days, or six sheep can eat the feed for 15 consecutive days. If it is required to eat all the sheep in 6 days, how many sheep do you need at least ?

Please answer on the answer sheet, the time is 4 minutes!

Example 3:

A basketball game starts at 14:00, and spectators are allowed to enter the arena at 13:30, but there are people waiting in line to enter the arena. Assuming that the number of spectators per minute is the same since the first spectator, if there are 3 entrances, there will be no queue at 13:45; if there are 4 entrances, there will be no queue at 13:40; what time will the first audience arrive?

Answer: Assuming that the number of spectators entering each entrance per minute is 1,

(1) The number of spectators per minute is: (3*15-4*10) /(15-10)=1

(2) The initial number of spectators is: 15* (3-1) = 30

(3) Then the time for the first spectator to arrive is: the time it takes for the spectator to enter the venue is 30/1=30 minutes, and the first spectator arrives 30 minutes forward at 13:30, which is 13:00.

Example 4

The art exhibition opens at 9:00, but there are people queuing up to enter the venue. From the time the first audience arrives, if the audience is the same every minute, if there are three entrances, there will be no more audiences at 9:09; if there are five entrances, there will be no more audiences at 9:5. Find the time when the first audience arrives?

Please answer on the answer sheet, the time is 4 minutes!

Same Examples in Example-problem Pairs Condition

Example 1:

A pasture is covered with grass, which grows at a uniform speed every day. This pasture can feed 10 cows for 20 days , or 15 cows for 10 days. How many days can 25 cows eat?

Answer: Suppose that the daily grass consumption of each cow is 1,

(1)The daily growth rate of grass is: (10*20-15*10)/(20-10)=5.

(2)The initial grass amount is: (10-5) * 20=100.

(3)Suppose 25 cows can eat for x days: (25-5) * x=100,x=5.

Example 2:

A pasture is covered with grass, which grows at a uniform speed every day. This pasture can feed 10 cows for 20 days , or 15 cows for 10 days. How many days can 25 cows eat?

Please answer on the answer sheet, the time is 4 minutes!

Example 3:

A basketball game starts at 14:00, and spectators are allowed to enter the arena at 13:30, but there are people waiting in line to enter the arena. Assuming that the number of spectators per minute is the same since the first spectator, if there are 3 entrances, there will be no queue at 13:45; if there are 4 entrances, there will be no queue at 13:40; what time will the first audience arrive?

Answer: Assuming that the number of spectators entering each entrance per minute is 1,

(1) The number of spectators per minute is: (3*15-4*10) /(15-10)=1

(2) The initial number of spectators is: 15* (3-1) = 30

(3) Then the time for the first spectator to arrive is: the time it takes for the spectator to enter the venue is 30/1=30 minutes, and the first spectator arrives 30 minutes forward at 13:30, which is 13:00.

Example 4:

A basketball game starts at 14:00, and spectators are allowed to enter the arena at 13:30, but there are people waiting in line to enter the arena. Assuming that the number of spectators per minute is the same since the first spectator, if there are 3 entrances, there will be no queue at 13:45; if there are 4 entrances, there will be no queue at 13:40; what time will the first audience arrive?

Please answer on the answer sheet, the time is 4 minutes!

Different Examples in Example-problem Pairs Condition

Example 1:

A pasture is covered with grass, which grows at a uniform speed every day. This pasture can feed 10 cows for 20 days , or 15 cows for 10 days. How many days can 25 cows eat?

Answer: Suppose that the daily grass consumption of each cow is 1,

(1)The daily growth rate of grass is: (10*20-15*10)/(20-10)=5.

(2)The initial grass amount is: (10-5) * 20=100.

(3)Suppose 25 cows can eat for x days: (25-5) * x=100,x=5.

Example 2:

As the weather gets colder, the grass on the pasture decreases at a uniform speed every day. The grass can feed 20 cows for 5 days, or 16 cows for 6 days. How many days can 11 cows eat?

Please answer on the answer sheet, the time is 4 minutes!

Example 3:

Due to the dry weather, the village committee decided to pump the remaining water from the reservoir to irrigate the farmland. The water added to the reservoir is increased at a uniform rate every day. According to the calculation, if 20 pumps are used to pump water fully, the water in the reservoir can be used for 5 weeks; If 16 pumps are used, the water in the reservoir can be used for 6 weeks; If 11 pumps are used, how many weeks can the water in the reservoir be used?

Answer: Suppose the weekly pumping capacity of each pump as 1,

(1) Suppose the weekly evaporation capacity as x: 20 * 5+5x=16 * 6+6x, x=4
(2) Suppose the initial water volume of the reservoir as: 20 * 5+5 * 4=120
(3) Suppose the water available in the reservoir as x weeks: x * (11+4)=120, x=8

Example 4:

There are three grasslands, covering an area of 4 hectares, 8 hectares and 10 hectares. The grass on the grassland is as thick and grows as fast. The first grassland can feed 24 cows for 6 weeks, and the second grassland can feed 36 cows for 12 weeks. How many weeks can the third grassland feed 50 cows?

Please answer on the answer sheet, the time is 4 minutes!

Appendix II Questionnaire

Thank you very much for participating in this experiment! After completing the basic information and filling in the first part, please follow the instructions carefully. If you do not understand, you can consult our staff before proceeding.

Basic Information

Name: ____ Major: _______ Age: ________ Grade: ________Gender: _______

First Part

1. Do you know the problem of "cow eating grass"? ________________
2. (Optional) If you know, where did you learn about it? ______________________
3. (Optional) Are you familiar with the calculation formula for the problem of "cow eating grass"? Please rate the level of familiarity, with 1 being very unfamiliar and 5 being very familiar.

1 2 3 4 5

Please stop reading below and follow the computer instructions before proceeding to complete the next few sections! ! !

The second part of the study

Formula sheet:

British scientist Newton proposed "the problem of cow grazing", that is, cows graze on the pasture, and the grass on the pasture grows continuously and uniformly. The "cow grazing" problem in the same pasture is formulated as:

$$\text{Grass growth rate} = \frac{(\text{grazing speed } 1 \times \text{time } 1 - \text{grazing speed } 2 \times \text{time } 2)}{(\text{time } 1 - \text{time } 2)}$$

$$\text{Initial grass amount} = (\text{grazing speed} - \text{grass growth speed}) \times \text{time}$$

(1) Example 2 (refer to the formula)

Answer:

Example 4 (refer to the formula)

Answer:

(2) Mental Effort Rating Scale

Please make a 9-point scale of the above learning content, that is, the mental effort value you put into solving the problem, where 1-9 represents 1.Very, very low effort; 2. Very low effort; 3. Low effort; 4. Slightly lower effort; 5. General effort; 6. Slightly higher effort; 7. High effort; 8. Very high effort; 9. Very, very high effort.

1 2 3 4 5 6 7 8 9

Part 3 Formal Test

Question 1:(refer to the formula)

Answer:

Question 2:(refer to the formula)

Answer:

(2) Mental Effort Rating Scale

Please make a 9-point scale of the above learning content, that is, the mental effort value you put into solving the problem, where 1-9 represents 1.Very, very low effort; 2. Very low effort; 3. Low effort; 4. Slightly lower effort; 5. General effort; 6. Slightly higher effort; 7. High effort; 8. Very high effort; 9. Very, very high effort.

1 2 3 4 5 6 7 8 9
